# Editorial: Community series in biology of C-reactive protein, volume II

**DOI:** 10.3389/fimmu.2025.1751020

**Published:** 2025-12-18

**Authors:** Alok Agrawal, Yi Wu

**Affiliations:** 1 Department of Biomedical Sciences, College of Medicine, East Tennessee State University, Johnson City, TN, United States; 2 MOE Key Laboratory of Environment and Genes Related to Diseases, School of Basic Medical Sciences, Xi’an Jiaotong University, Xi’an, China

**Keywords:** C-reactive protein, amyloidosis, pneumococcal infection, Fc receptors, biomarkers of inflammation

C-reactive protein (CRP) is an evolutionarily conserved protein with pattern recognition receptor-like activities (1). Experiments performed *in vitro* and employing mouse models of human diseases have revealed that CRP possesses anti-bacterial, anti-atherosclerotic, anti-arthritic and anti-amyloidogenic activities (2–8). In addition to executing host-defense functions, CRP also serves as a biomarker of inflammation (9). This Research Topic includes nine articles: four of these articles report latest findings on the structure-function relationships of CRP and the other five articles cover the developments in the clinical utility of CRP.

CRP exists and is active in three different structural conformations: native pentameric CRP, monomeric CRP (mCRP) and intermediate or non-native or transitional or modified or loosened pentameric CRP (pCRP*) (10). The abbreviation CRP represents native pentameric CRP. Potempa et al. reviewed the literature on the three forms of CRP and re-evaluated their roles in the tissue repair processes and thereby resolving some controversies on the structure-function relationships of CRP.

It has been shown previously that CRP binds to Fcγ receptors (FcγR); neither the complexing of CRP with a ligand nor a conformational change in the native pentameric structure of CRP was required for CRP to bind to FcγRs (11, 12). Henning et al. thoroughly investigated the CRP-FcγR interactions by employing both CRP and mCRP in both free and immobilized forms. They found that both forms of immobilized CRP engaged both the activating and inhibitory FcγRs. Activation of FcγRs by fluid-phase CRP was considerably lower than with immobilized CRP but was enhanced in the presence of streptococci. Immobilization of CRP exposed mCRP epitopes, suggesting that pCRP*, not CRP, is the major FcγR-activating conformation.

The primary ligand-binding specificity of CRP is for phosphocholine (PCh)-containing substances such as cell wall C-polysaccharide of pneumococci. CRP has been shown to protect mice against lethal pneumococcal infection but only when CRP is administered to mice during the early stages of infection. Employing CRP-deficient mice in pneumococcal infection experiments, Agrawal et al. found that CRP was protective against late-stage infection also; however, the protection against late-stage infection involved structural changes in CRP and subsequent binding of CRP to PCh and of structurally altered CRP to both PCh and amyloids present on the bacterial surface. In addition, their data also suggested that the amyloid-binding protein, serum amyloid P component, cooperated with CRP in reducing bacteremia and bacterial load in mice infected with pneumococci. The PCh-binding function of CRP has been conserved throughout evolution from arthropods to humans. However, it was not known whether the amyloid-binding function of structurally altered CRP has also been evolutionarily conserved. Agrawal et al. isolated arthropod CRP from American horseshoe crab *Limulus polyphemus* (Li-CRP) and investigated the anti-amyloidogenic activity of Li-CRP. They report that Li-CRP binds to amyloids and prevents the formation of amyloid fibrils, and unlike human CRP, Li-CRP does not require any changes in its overall structure to bind to amyloids. Their data further suggested that a variety of Li-CRP molecules of different subunit compositions were present in *Limulus* hemolymph, raising the possibility that the presence of various Li-CRP species in hemolymph facilitates the recognition of a range of proteins with differing amyloidogenicity. It was concluded that the binding of CRP to amyloids is also an ancient function of CRP. In invertebrates, the amyloid-binding function of CRP can protect the host from toxicity caused by amyloidogenic and pathogenic proteins. In humans, the amyloid-binding function of CRP can protect against inflammatory diseases in which the host proteins are ectopically deposited on either host cells or foreign cells in an inflammatory milieu since immobilized proteins may expose amyloid-like structures after deposition at places where they are not supposed to be.

Since the serum level of CRP rises in inflammatory states, serum CRP is used as a non-specific biomarker of inflammation. In this Research Topic, there are five reports on the use of CRP as a co-biomarker for specific diseases. Chen et al. investigated whether CRP could be used as a co-biomarker to improve the prediction of venous thromboembolism in patients with bladder cancer. They found that the combined model of elevated CRP and elevated D-Dimer levels offers superior predictive performance. Chen et al. investigated whether CRP could be used as a co-biomarker for predicting severity in acute pancreatitis. They report that the ratio of serum CRP to serum calcium levels is a novel biomarker for predicting severity in acute pancreatitis in their retrospective cross-sectional study. However, further multicenter prospective cohort studies are needed to confirm its clinical utility. Huang et al. performed a cross-sectional study and investigated whether CRP could be used as a co-biomarker for the prevalence of hyperuricemia in adults with diabetes or prediabetes. They found that an elevated CRP to high-density lipoprotein cholesterol ratio was significantly associated with a higher risk of hyperuricemia in adults with diabetes or prediabetes. Wu et al. investigated the association of CRP with hepatic fibrosis in US and Chinese patients with metabolic dysfunction-associated steatotic liver disease (MASLD) and assessed its predictive efficacy. They found a significant correlation between elevated CRP levels and increased risk of fibrosis and cirrhosis in US MASLD patients and between elevated CRP levels and hepatic fibrosis in Chinese MASLD patients. Finally, Xu et al. reviewed the published literature and performed meta-analysis of the data to compare the diagnostic accuracy of resistin and CRP levels for sepsis in neonates and children. They found that both CRP and resistin levels could be used as biomarkers for detecting pediatric and neonatal sepsis.

In summary, recent data indicate that the native pentameric structure of CRP is flexible and that the structural changes in CRP is a key mechanism for CRP to be protective against inflammatory diseases. Appropriately designed animal models can be used to further understand the conformation-dependent actions of CRP *in vivo* (1). Potempa et al. provides a review of the shortcomings of the currently available diagnostic tests for CRP and highlight the need for change in how CRP is currently utilized in clinical practice.
